# SPECT and PET imaging of Alzheimer’s disease revisited: from biomarkers to artificial intelligence-based prediction

**DOI:** 10.1007/s12149-026-02194-0

**Published:** 2026-03-26

**Authors:** Ioannis Tsougos, Varvara Valotassiou, Dimitra Tsivaka, Maria Satra, George Angelidis, John Papatriantafyllou, Emmanouil Panagiotidis, Efthimios Dardiotis, George Hadjigeorgiou, Panagiotis Georgoulias

**Affiliations:** 1 Medical Physics Department, Faculty of Medicine, University of Thessaly, Panepistimiou 3, Biopolis, 41500 Larisa, Greece; 2 Department of Nuclear Medicine, Faculty of Medicine, University of Thessaly, University Hospital of Larissa, Larissa, Greece; 3 Faculty of Public and One Health, University of Thessaly, Karditsa, Greece; 4 IASIS Community Medical Center for the Elderly, Athens, Greece; 5 Department of Neurology, University Hospital of Larissa, University of Thessaly, Larissa, Greece; 6 Medical School, University of Cyprus, Nicosia, Cyprus

**Keywords:** Alzheimer’s disease, Positron Emission Tomography, Single-Photon Emission Computed Tomography, Artificial Intelligence

## Abstract

In Alzheimer’s disease (AD), PET and SPECT enable in-vivo imaging of β-amyloid, tau, cerebral metabolism, and neuroinflammation. However, classical interpretation, including visual reads, ROI summaries, and SUVR thresholds, remains limited by reader variability, dependence on reference regions, and cross-site heterogeneity. Building upon our previous review on SPECT and PET imaging in AD, this article revisits molecular neuroimaging through the lens of artificial intelligence (AI), integrating advances from radiomics and classical machine learning to deep learning that support more quantitative and predictive use of PET/SPECT. Methods are organized by clinical objective, including diagnostic and differential classification, segmentation for region-wise measurement, automated quantification, image enhancement and reconstruction (attenuation correction, denoising, super-resolution, low-dose/short-scan recovery), and prognostic modeling (conversion and cognitive decline). We summarize key data resources, benchmarking, and standardization /harmonization strategies that improve generalization across scanners and tracers. Finally, we outline practical requirements for translation: models should provide well-calibrated probabilities, indicate when predictions are uncertain, offer outputs consistent with AD-relevant biology, report performance across relevant subgroups, and follow transparent reporting standards with clinically usable outputs, supporting earlier detection and more consistent monitoring in AD.

## Introduction

Alzheimer’s disease (AD) is the leading cause of dementia worldwide and continues to represent a major challenge for healthcare systems and societies. Its neuropathological hallmarks, extracellular deposition of beta-amyloid (Aβ) plaques, intracellular neurofibrillary tangles (NFTs) of hyperphosphorylated tau protein, and associated neuroinflammatory responses, precede clinical symptom onset by years or even decades [1, 2]. Molecular nuclear imaging with positron emission tomography (PET) and single-photon emission computed tomography (SPECT) allows the in vivo visualization of these pathological processes. In our previous review [3], we highlighted how radiotracers targeting Aβ, tau, and neuroinflammation had already begun to reshape the diagnostic framework of AD, offering valuable biomarkers for early detection and disease monitoring.

Since then, further advances in radiopharmaceuticals and imaging protocols have improved specificity and quantification. However, several challenges remain. Conventional approaches to image interpretation, including visual reads and semi-quantitative region-of-interest (ROI) analyses, suffer from inter- and intra-observer variability, limited sensitivity to subtle or diffuse changes, and lack of cross-center standardization [4–6]. These limitations have stimulated growing interest in the application of artificial intelligence (AI) to neuroimaging, particularly in the domains of image processing, pattern recognition, and predictive modeling.

AI techniques, ranging from classical machine learning (ML) to deep learning (DL) and radiomics, are increasingly used to extract high-dimensional features from PET and SPECT data, offering enhanced disease characterization beyond human visual capacity [7–10]. Notably, AI can facilitate objective, reproducible quantification of pathological burden (e.g., amyloid or tau load) [11, 12], support automated segmentation of brain structures, and integrate multimodal inputs, including genetic, cognitive, and clinical parameters, into individualized risk prediction models [13, 14]. Furthermore, recent studies have demonstrated the utility of AI for identifying individuals at high risk for progression from mild cognitive impairment (MCI) to AD [9, 15], stratifying patients in clinical trials [16], and assessing treatment response with increased sensitivity [17].

In this review, we aim to build upon our previous work by presenting a state-of-the-art synthesis of how artificial intelligence is transforming nuclear neuroimaging in Alzheimer’s disease. We focus on AI applications in PET and SPECT imaging, from tracer quantification and spatial pattern analysis to prognostic modeling, critically appraise methodological and translational challenges, and outline priorities for integrating robust, explainable, and standardized AI tools into clinical nuclear medicine and AD research.

## Advances in PET/SPECT tracers and limitations of classical quantification

In recent years, the development of novel radiotracers targeting the pathophysiological hallmarks of Alzheimer’s disease has significantly advanced the diagnostic capabilities of PET and SPECT imaging. Aβ tracers, ^11^C-PiB, ^1^⁸F-florbetapir, ^1^⁸F-florbetaben and ^1^⁸F-flutemetamol, show high sensitivity/specificity for plaque detection [18–20]. Tau agents, ^1^⁸F-AV-1451 (flortaucipir), ^1^⁸F-MK-6240, ^11^C-PBB3, enable in-vivo assessment of neurofibrillary tangle (NFT) supporting Braak-like staging and differential diagnosis [21–23]. TSPO-targeted ligands, ^11^C-PK11195, ^1^⁸F-DPA714, ^11^C-PBR28, visualize microglial activation [24]. Traditional visual assessments are inherently subjective and highly dependent on reader expertise, especially in early-stage or borderline cases [6]. Semi-quantitative methods, such as SUVRs, aim to mitigate this subjectivity by comparing tracer uptake in target regions to reference regions (e.g., cerebellum or pons). However, these approaches rely on assumptions about the stability of reference regions, lack universally accepted cut-off values, and do not fully capture complex regional uptake patterns or diffuse pathology [25–27].

ROI-based approaches may overlook subtle extra-regional changes and show variable reproducibility across sites due to preprocessing, spatial normalization, and region-definition differences [28, 29]. Centiloid improves inter-study comparability for amyloid PET, but integrating multi-tracer datasets and heterogeneous hardware remains challenging [26]. Automated tools (SPM, 3D-SSP, eZIS) project data to standardized templates to aid objectivity, yet still assume normality, adapt poorly to heterogeneous populations, and often require expert oversight [30, 31].

Collectively, reader subjectivity, cross-site/scanner variability, reference-region assumptions, partial-volume effects, and ROI-bounded analyses, these constraints motivate advanced computation. AI, particularly machine learning and deep learning, offers high-dimensional quantification and robust pattern recognition, cross-site harmonization, low-dose/short-scan recovery, and multimodal fusion (PET/SPECT with MRI, cognition, genetics) for individualized risk prediction. In the next section, we map these problems to specific AI solutions and methodological foundations.

## AI rationale, methods, and data foundations

Before focusing on Alzheimer’s disease specific applications, it is helpful to briefly outline how artificial intelligence is used within PET and SPECT imaging workflows to better understand how, and at which points, AI is subsequently tailored to address Alzheimer’s disease-related questions in PET and SPECT, including diagnosis, staging, and prognosis.

### AI fundamentals for PET/SPECT

In nuclear neuroimaging, AI methods can be broadly divided into classical ML and DL approaches, which differ mainly in how imaging information is represented and analyzed.

Classical ML relies on predefined, human-designed features extracted from PET or SPECT images, such as regional standardized uptake values (SUV/SUVR), uptake ratios, or radiomics descriptors that quantify texture, intensity, and shape. These features are then analyzed using established statistical or ML models (e.g., support vector machines etc.). Such approaches have been widely used in FDG, amyloid, and tau PET to support diagnosis and prognosis in Alzheimer’s disease, but their performance depends strongly on feature selection and a priori assumptions about relevant regions [17, 32].

Deep learning, in contrast, learns relevant image representations directly from the data, without requiring explicit feature engineering. Convolutional neural networks (CNNs), the most commonly used DL models in medical imaging, process images or volumes through successive layers that automatically capture spatial patterns of tracer uptake, including subtle and distributed changes characteristic of AD. This ability to model complex, multiregional patterns explains why DL approaches often outperform ROI-based analyses, particularly in heterogeneous or early disease stages [10, 14]. CNNs currently constitute the core architecture for most PET/SPECT AI pipelines for classification (e.g., AD vs MCI vs cognitively normal), automated quantification, and differential diagnosis. They can naturally handle three-dimensional data, multi-channel inputs (e.g., multiple tracers or PET combined with MRI), and voxel-wise context.

Related DL architectures address complementary needs: autoencoders, including variational autoencoders (VAEs), learn compact representations of images in an unsupervised manner and are particularly useful for denoising, anomaly detection, or low-count data scenarios [33, 34]. Transformer-based models, originally developed for sequence modeling, are increasingly adopted in PET for reconstruction, denoising, and multimodal fusion, as they can capture long-range dependencies that are difficult for standard CNNs alone [35, 36]. More recently, graph neural networks (GNNs) have been explored to model metabolic or functional connectivity derived from PET, or to integrate imaging with clinical and demographic information, enabling network-level representations of AD pathology [37, 38].

For clinical clarity, AI methods in PET and SPECT for Alzheimer’s disease are best organized by clinical objective rather than by algorithmic class. Most applications fall into five interrelated task categories:

- *Classification*, supporting diagnostic decisions (AD vs MCI vs controls, or differential diagnoses), typically using CNNs or transformer models applied to FDG, amyloid, or tau PET, often augmented with clinical or cognitive variables [39, 40].
- *Segmentation*, which automatically delineates brain regions or pathological targets and serves as a foundation for region-wise quantification, commonly implemented with U-Net-type CNNs or transformer variants [32].
- *Quantification*, where DL models estimate standardized metrics such as SUVR or Centiloid directly from images, or approximate parametric information when full kinetic modeling is impractical, reducing reliance on reference-region assumptions and strict scan timing [41].
- *Synthesis*, encompassing DL-based attenuation correction (AC) without CT/MR, pseudo-modality generation (e.g., pseudo-CT), and low-dose or short-scan reconstruction through denoising or super-resolution, with the aim of reducing radiation dose or acquisition time while preserving quantitative accuracy [36, 42, 43].
- *Prognostic modeling*, where PET-derived features (alone or combined with clinical and cognitive data) are used to predict cognitive decline, supporting individualized risk assessment and trial enrichment [40, 44].

### Data sources and validation in PET/SPECT AI

AI models in PET and SPECT for AD are typically developed using large, well-curated neuroimaging datasets, most commonly ADNI [45, 46], AIBL [47], OASIS-3 [48], and DIAN [49], which provide standardized acquisitions, longitudinal follow-up, and validated clinical labels. Open healthy-control PET datasets are comparatively rare but useful for normalization and augmentation (e.g., CERMEP-IDB-MRXFDG, a public PET/MR database of normal brain FDG-PET scans) [50], whereas truly open perfusion SPECT dementia datasets remain scarce. These resources support tasks such as diagnosis, staging, and prognosis, but differ in scanners, tracers, demographics, and follow-up schemes, introducing domain variability that directly affects AI generalization.

Robust benchmarking requires subject-wise data splits, careful handling of repeated scans, and evaluation on external datasets or sites to avoid optimistic bias [51]. Transparent reporting frameworks such as TRIPOD-AI (the AI extension of the Transparent Reporting of a multivariable prediction model for Individual Prognosis Or Diagnosis guideline) support reproducibility by standardizing how datasets, preprocessing, data splits, calibration, and validation hierarchies are described [52].

When data sharing is restricted, federated learning enables multi-center model training without centralizing raw images [53–55]. Across both PET and SPECT, quantitative comparability further depends on harmonization strategies such as EARL accreditation (for FDG PET) and Centiloid scaling (for amyloid PET), which reduce scanner- and tracer-related variability prior to or alongside AI modeling [56].

### Image preparation and quantitative standardization in PET/SPECT AI

*Preprocessing*: Reliable PET and SPECT quantification depends on appropriate preprocessing. Attenuation correction is essential in PET/MR, bone and air segmentation and artifact handling are major sources of variability that directly affect SUVs/SUVRs and downstream analyses [42]. Established AC approaches include template-, atlas-, segmentation-, and learning-based methods, while recent deep-learning techniques allow CT-less AC directly from emission or MR data [57]. Head motion degrades image quality and increases variability in amyloid and tau PET [58], so data-driven frame- or list-mode motion correction improves visual interpretation and stabilizes regional measures [59]. Additionally, spatial normalization choices (e.g., SPM-based workflows) influence voxel-wise maps and regional summaries [31].

*Quantification and reference scaling*: Standardized uptake values (SUV/SUVR) remain the most common quantitative measures in clinical and research practice. However, reference-region selection (e.g., whole cerebellum, pons/brainstem) and acquisition timing significantly affect absolute SUVR values and longitudinal change, highlighting the need for transparent methodological reporting [60]. For amyloid PET, the Centiloid framework expresses uptake on a standardized 0—100 scale, improving comparability across tracers and studies [26]. Consensus and regulatory documents support its use while noting residual variability related to processing pipelines and tracer choice [61].

*Harmonization and domain adaptation*: Differences between scanners and reconstruction settings require harmonization. The EANM/EARL accreditation program defines performance standards that reduce inter-system variability and facilitate multicenter comparability, particularly for FDG PET [56, 62].Post-hoc statistical approaches (e.g., ComBat) and DL-based harmonization further reduce site- and tracer-related variability, in some cases improving regional sensitivity and prediction of cognitive decline beyond conventional scaling [63–66].

When MR or CT data are available, cross-modality supervision and transfer learning (e.g., pseudo-CT for AC, PET-MRI synthesis) support reconstruction, denoising, and domain alignment [67].

**Fig. 1:**
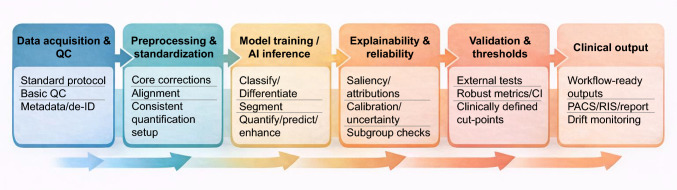
End-to-end analytical AI pipeline for PET and SPECT imaging in Alzheimer’s disease, spanning standardized data acquisition and preprocessing, task-specific AI inference, explainability and reliability assessment, validation and calibration, and deployment as workflow-ready clinical outputs

Figure 1 shows a conceptual end-to-end analytical AI pipeline for PET and SPECT imaging in Alzheimer’s disease, illustrating the sequential steps from standardized data acquisition and preprocessing, through task-specific AI inference and reliability assessment, to validation, calibration, and integration into clinical workflows.

## AI in SPECT for Alzheimer’s disease

### Perfusion SPECT

Early machine-learning work showed that multivariate pattern analysis on cerebral perfusion SPECT can separate AD from controls and stratify MCI, sometimes approaching FDG-PET performance depending on the classifier and cohort composition [68–70]. In an ADNI-anchored study that also included an external perfusion SPECT cohort (^99m^Tc-HMPAO), support-vector approaches achieved balanced performance for AD vs normal controls, and stable vs prodromal MCI, with SPECT performing competitively in some settings but also showing more false positives in stable MCI, highlighting the importance of external validation and calibration to clinical priors [69].

Recent deep-learning studies shift from handcrafted features and z-maps to end-to-end CNNs trained on perfusion SPECT volumes [71–73]. A notable example is Ni et al. (2021), who leveraged two-stage transfer learning, a model first trained on thousands of ADNI FDG-PET scans was fine-tuned on a modest 99mTc-ECD SPECT dataset (n = 247) [71]. This approach achieved AUC ≈ 0.90 for AD vs normal controls and, importantly, produced a reasonable continuous correlate of cognition (Mini-Mental State Examination (MMSE), R^2^≈ 0.71), suggesting the DL latent space captures disease severity beyond binary labels.

Beyond binary diagnosis, CNNs on z-score/3D-SSP representations have been used to encode disease-specific topographies and improve discrimination in dementia differentials (e.g., Lewy Body Dementia-LBD vs AD via cingulate-island–related perfusion patterns) [74]. While focused on differential diagnosis, these works underscore how template-space projections and z-maps can serve as inputs or augmentation to DL models when native 3D training data are scarce [31, 74]. In a CAD-style precursor, Imabayashi et al. used eZIS-generated, template-space perfusion SPECT z-score maps and optimized VOI-based cingulate-island-sign ratios to discriminate DLB from AD, illustrating how automated quantitative pipelines anticipated later ML/DL workflows [75].

Finally, clinic-grade pipelines increasingly pair standard SPECT preprocessing (normalization, spatial registration) with 2D/3D CNNs (e.g., Inception-based) and interpretation tools (Grad-CAM) to deliver transparent outputs and region-level explanations, even extending to AD vs vascular dementia (VaD) tasks on ECD SPECT [31, 72, 74, 76]. With careful transfer learning and/or z-score augmentation, DL on perfusion SPECT achieves competitive AD discrimination and begins to map onto cognitive severity, provided external, site-diverse testing is performed [71].

### Low-count protocols and reconstruction-aware DL

SPECT’s lower photon statistics motivate AI for dose/time reduction and quantitative fidelity. Although much of the algorithmic maturation occurred in PET and cardiac SPECT, the principles translate to brain perfusion SPECT:

*Deep denoising* for low-dose/short-scan SPECT has demonstrated marked gains in task performance and quantitative accuracy in myocardial perfusion cohorts (projection-domain approaches often outperform image-domain), establishing best practices for projection-aware networks and task-based evaluation that are directly applicable to brain protocols [77, 78].

*Attenuation correction* without CT: CNNs trained to generate AC-equivalent brain SPECT from non-AC inputs have shown promising quantitative recovery, reducing the dependency on SPECT/CT hardware, highly relevant for legacy systems and dose-sensitive workflows [79, 80].

*Direct, DL-based reconstruction* (sinogram to image) such as SPECTnet demonstrates feasibility for learning the imaging operator end-to-end, potentially enabling count-efficient and artifact-aware recon that preserves clinical contrast for downstream quantification or classification. While demonstrated broadly, these architectures offer a blueprint for brain perfusion pipelines aiming to shorten scans without sacrificing SUVR-like metrics [81].

*Brain-specific image-quality enhancement* on 99mTc-ECD SPECT has also been reported with DL, supporting practical gains in contrast/noise that can stabilize automated reads. These reports are emerging from nuclear medicine meetings and early translational series [82].

To responsibly deploy low-count SPECT AI, task-based endpoints (diagnostic accuracy, reader AUC, region-wise perfusion bias) should accompany image-quality metrics, and projection-domain or reconstruction-aware learning tends to generalize better across protocols.

### Cross-vendor calibration and harmonization

Domain shift, scanner models, collimators, reconstruction kernels, and site-specific preprocessing, remains a primary threat to generalization for SPECT AI. In multicenter ECD SPECT studies, pipelines commonly rely on template-space normalization and resampling to reduce inter-system variability [72, 73]. However, these steps mitigate rather than eliminate scanner/collimator and reconstruction differences, underscoring the value of explicit harmonization or domain adaptation when training across vendors and tracers.

Statistical harmonization (e.g., ComBat) and deep domain adaptation are well studied in neuroimaging and increasingly applied around AD imaging to correct scanner/site effects [65]. Although many examples come from MRI/PET, the methods are modality-agnostic and suitable for SPECT [83, 84]. Reporting should explicitly document site composition and reconstruction settings, and wherever feasible, include a held-out external site for performance estimation. When SPECT/CT is unavailable or AC varies, DL-based AC can serve as a standardization layer prior to quantification/AI inference, narrowing inter-site bias.

### Severity prediction and cognitive correlates

Beyond classification, several studies point to continuous severity estimation and cognition linkage from perfusion SPECT. Ni et al. (2021) reported a continuous MMSE prediction from ECD SPECT with R^2^≈ 0.71, suggesting that DL features track clinical severity and could support staging or monitoring [71]. In parallel, CNN pipelines for AD vs VaD have begun to incorporate explainability (Grad-CAM) to localize perfusion deficits, a step toward clinically interpretable severity maps and hypothesis-driven correlation with neuropsychology [72].

As low-count/reconstruction-aware DL matures, test–retest stability and calibration of regional perfusion metrics will become critical so that longitudinal changes reflect biology rather than domain shift. Lessons from PET DL denoising/reconstruction, uncertainty estimation, calibration curves, and federated training, are directly relevant to SPECT severity modeling [36, 85–87].

To consolidate the above findings, Table 1 summarizes representative AI studies on brain perfusion SPECT spanning end-to-end CNNs, transfer learning, z-score/3D-SSP–based approaches, and classical MVPA comparisons across sites and tracers.

**Table 1:** Representative studies about AI in SPECT for Alzheimer’s disease

Study (Year)	Task	Modality / tracer	Model / features	Cohort (N), sites	External test	Key results
Ni et al., 2021	AD vs CN, MMSE regression	Brain perfusion SPECT, Tc‑99 m‑ECD	Two-stage transfer learning (Inception‑v3): ImageNet/ADNI FDG pretrain → ECD fine‑tune; Grad‑CAM	ECD SPECT n = 247 (AD 113, CN 134) train/val n = 197 (AD 91, CN 106) internal test n = 50 (AD 22, CN 28). ADNI FDG-PET pretrain ~ 1,000 scans	Internal held-out test, cross-modality transfer (ADNI FDG-PET → ECD SPECT)	AD vs CN AUC ≈ 0.90 with FDG pretraining. MMSE regression improved (R^2^ ≈ 0.71 train; ≈ 0.22 test); DL heatmaps show temporo-parietal focus
Ni et al., 2024	AD vs VaD (differential) + explainability	Brain perfusion SPECT, Tc‑99 m‑ECD	Inception‑v3 end‑to‑end + Grad‑CAM, SPM analysis for comparison, template normalization & resampling	Multicenter (4 medical centers) database n = 197 (AD 112, VaD 85). train/val n = 177 (AD 103, VaD 74). internal test n = 20 (AD 9, VaD 11)	Internal independent test (20 images)	fivefold mean: AUC 0.95 (0.73–1.00); Grad‑CAM vs SPM correspondence, detailed preprocessing
Iizuka et al., 2019	DLB vs AD using 3D‑SSP z‑maps	Perfusion SPECT (I‑123‑IMP), z‑score (3D‑SSP) inputs	CNN on z‑maps, cingulate‑island sign (CIS) localization with DL	Single-center: training/validation 80 DLB, 80 AD, 80 NL. test 20/20/20 (3D-SSP images)	No	Accuracy ~ 89% for DLB vs AD; CIS captured by CNN on 3D‑SSP
Lien et al., 2023	Cognitive impairment severity classification	Brain perfusion SPECT (Tc-99 m-ECD)	Comparative CNN study (MobileNetV2, NASNetMobile, VGG16, Inception V3, ResNet), transfer learning examined	99 subjects, 4,711 images (single center)	No (single-center internal test set)	Demonstrated effectiveness of transfer learning, established baseline performance across CNN families for SPECT-based severity staging
Katako et al., 2018	AD vs CN, prodromal AD vs stable MCI, multi‑dx	HMPAO SPECT vs ADNI FDG‑PET	SVM/MVPA vs GLM/SSM‑PCA	ADNI FDG-PET n = 763. external perfusion SPECT (Tc-99 m-HMPAO) MCI n = 38 (Busan, > 3y follow-up). Plus other clinical datasets	Yes (multi‑site)	SPECT competitive to FDG under some SVM configs, more FP in stable MCI; site/classifier dependence

**Fig. 2:**
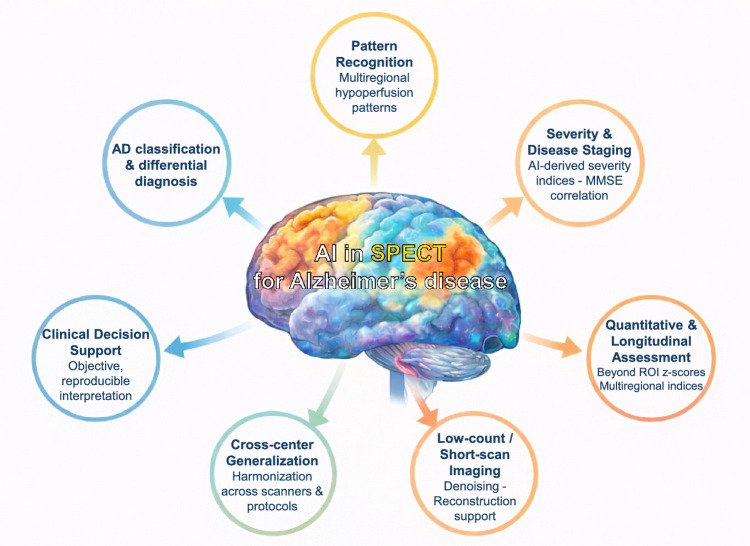
Overview of key artificial intelligence applications in perfusion SPECT imaging for Alzheimer’s disease. AI methods support data-driven recognition of multiregional hypoperfusion patterns, diagnostic and differential classification, quantitative enhancement and severity staging, low-count or short-scan imaging, harmonization across scanners and protocols, and objective clinical decision support

Figure 2 summarizes the main applications of artificial intelligence in SPECT imaging for Alzheimer’s disease, highlighting data-driven pattern recognition, diagnostic and differential classification, quantitative enhancement beyond conventional z-score approaches, severity and disease staging, low-count/short-scan imaging, cross-center generalization, and clinical decision support.

## AI in PET for Alzheimer’s disease

### FDG-PET

Deep learning has become the dominant approach for FDG-PET pattern recognition in AD, consistently separating AD from cognitively normal (CN) participants and stratifying MCI [88–90]. A recent systematic review/meta-analysis pooling 36 DL–FDG studies reported pooled AUCs of 0.98 (AD vs CN), 0.95 (AD vs MCI) and 0.94 (MCI vs CN), while also emphasizing between-study heterogeneity and the need for external validation and reporting rigor [88].

Beyond pure diagnosis, FDG-based DL models are increasingly used in prognostic settings [40, 44, 91]. For example, a model originally trained for AD diagnosis was shown to add prognostic value for cognitive decline beyond clinical covariates in pre-dementia cohorts (MCI/CN) [44]. Classic multivariate FDG metrics such as the Hypometabolic Convergence Index (HCI) remain relevant as baselines and for network-level interpretation, and they have been linked to clinical progression in ADNI [92].

Network-aware or connectomic analyses with FDG are also gaining traction. Graph- and covariance-based approaches model metabolic connectivity and can complement voxel-wise classifiers. Reviews and studies show how metabolic covariance networks and graph learning frame AD-typical network disruptions [93, 94]. Early DL work has also explored 3D CNNs for multi-diagnosis classification including AD vs frontotemporal dementia (FTD) using FDG volumes, highlighting volumetric context for pattern recognition [39]. Finally, multimodal FDG pipelines that fuse imaging with cognitive/clinical data improve conversion prediction (MCI to AD) and risk stratification.

### Amyloid PET

End-to-end models can now infer amyloid positivity directly in native space and generalize across scanners and tracers without MRI. The AmyloidPETNet study tested on 8,476 scans from five datasets (ADNI, OASIS-3, Centiloid, SEABIRD, A4) and five tracers (FBP/FBB/FMT/NAV4694/PiB), achieving AUC ≥ 0.95 and performing well even in cognitively unimpaired A4 participants [95]. Attention maps aligned with cortical gray–white patterns expected for Aβ deposition.

For automated quantification, several DL approaches estimate SUVR or Centiloid with reduced preprocessing. A 3D-ResNet trained on PiB directly predicted Centiloid and generalized to four ^18^F tracers (NAV4694, FBB, FMT, FBP) without retraining, leveraging GAAIN reference datasets, an important step for cross-tracer standardization [12]. Other works explored DL SUVR estimation in native space (e.g., pretrained 2D/3D CNNs on FBP/FBB) and GAN-based pseudo-MRI to enable PET-only pipelines, reducing reliance on MRI segmentation [96, 97].

Dose and time reduction is increasingly feasible: ultra-low-dose amyloid PET/MR enhanced with DL produced diagnostic-quality images and preserved clinical utility, with follow-ups showing robustness under simultaneous or non-simultaneous MR [98, 99]. More generally, DL denoising/super-resolution methods validated in multi-site oncologic PET demonstrate non-inferiority at fourfold count reduction, a principle transferable to amyloid PET if task-based image quality is preserved [100]. Complementarily, Choi et al. showed that dual-phase ^1^⁸F-florbetaben PET can be exploited beyond the delayed amyloid read: early-phase (0–10 min) perfusion/ metabolism-proxy regional SUVRs combined with clinical variables (notably APOE) predicted delayed-phase Aβ positivity with 88.9% accuracy, supporting “multi-biomarker” extraction from a single tracer injection [101].

### Tau PET

DL has been used to leverage regional staging patterns from tau PET (e.g., flortaucipir, MK-6240) for group discrimination (CN vs MCI vs AD) and to better capture disease topology [14, 102–105]. A multimodal DL study on ^18^F-flortaucipir (ADNI) improved classification over unimodal inputs by integrating demographics and neuropsychology, suggesting that tau PET features contribute complementary staging information [14]. Methodological context on tracer performance and Braak-like distributions across flortaucipir and MK-6240 is provided by direct tracer comparisons [106].

A persistent challenge is off-target binding (e.g., choroid plexus/basal ganglia for flortaucipir). Emerging DL pipelines attempt normalization-free classification and domain-aware training to reduce sensitivity to off-target signal (conceptually applicable to AD staging) [107]. In parallel, DL is being explored for partial-volume/resolution enhancement to stabilize regional tau burden (e.g., DeepPVC and related CNN/cycle-consistent adversarial schemes), aiming to mitigate cortical spill-in/spill-out without labour-intensive segmentation [108, 109].

Finally, CNNs have been used to map Aβ–tau associations beyond linear models, hinting that DL can capture cross-pathology dependencies relevant to staging and prognosis [110].

### Multi-tracer and cross-tracer models

Generalization across tracers and sites is critical. AmyloidPETNet explicitly trained/evaluated across multiple datasets and five tracers in native space, reporting strong external performance, an effective tracer-agnostic strategy [95]. Complementarily, Centiloid-prediction DL trained on PiB and tested zero-shot on multiple ^18^F tracers demonstrates task-specific cross-tracer generalization for quantitative endpoints [12].

For settings where mixing tracers/sites introduces domain shift, DL-based harmonization is emerging (e.g., models that learn tracer-invariant embeddings or directly harmonize images for downstream tasks) [66]. These strategies align with statistical harmonization (e.g., Centiloid) but bypass heavy preprocessing and can be paired with external test sets for honest generalization estimates.

### Prognostic modeling

AI models using PET increasingly target individualized prognosis: predicting conversion (MCI to AD), cognitive decline, and time-to-event. Survival-style DL on longitudinal multimodal data shows that imaging-derived risk scores (often spearheaded by PET) can estimate conversion timelines more accurately than traditional baselines [111–113]. FDG-based DL has been shown to improve prediction of future cognitive decline beyond clinical data in pre-dementia groups, and FDG-neurocognitive fusion further boosts 3-year conversion prediction from MCI [40, 44]. Broader reviews also support PET-driven AI biomarkers for trial enrichment and risk stratification [114].

On the amyloid side, DL harmonization/aggregation of amyloid PET signals has been linked to risk of future decline in non-demented elders, complementing FDG-based risk models [66].

To summarize, Table 2 highlights representative AI in PET studies across FDG, amyloid, and tau, emphasizing: native-space, MRI-free amyloid classification and DL quantification that generalize, low-dose/short-scan recovery and harmonization, and prognosis (decline/conversion) with proper external validation and calibration.

**Table 2:** Representative studies about AI in PET for Alzheimer’s disease

Study (Year)	Task	PET target / tracer(s)	Model / key method	Cohort / external test	Key results
Fan et al., 2024	Screen for AD-related amyloid positivity across CN/MCI/AD, multi-tracer, MRI-free (native space)	Amyloid (FBP, FBB, FMT, NAV4694, PiB)	AmyloidPETNet (3D CNN, end-to-end native-space amyloid-positivity classifier)	8,476 scans; ADNI, OASIS-3, Centiloid, SEABIRD, A4; multi-tracer	For AD screening, AUC ≥ 0.95 across sites/tracers; strong performance in A4 cognitively unimpaired cohort
Yamao et al., 2024	Automated AD amyloid burden (Centiloid) estimation; train on PiB, zero-shot to multiple AD tracers	Amyloid (PiB train → NAV4694/FBB/FMT/FBP test)	3D ResNet-50 (native-space Centiloid-scale regression)	GAAIN datasets; PiB-trained 3D-ResNet-50 tested on 4 tracers	Centiloid vs standard: R^2^≈0.956; best PiB→NAV4694 transfer, supports cross-tracer AD quantification
Chen et al., 2022	DL restoration of ultra-low-dose amyloid PET for AD evaluation (PET/MR)	Amyloid (PET/MR)	U-Net–based CNN (2D, MR-guided ultra-low-dose amyloid PET enhancement)	Prospective PET/MR; simultaneous & non-simultaneous MR	Diagnostic-quality AD amyloid images at ultra-low dose, robust to simultaneous/non-simultaneous MR
Choi et al., 2024	DL harmonization of amyloid PET to predict AD-related cognitive decline in non-demented elders	Amyloid (multi-tracer)	Revised residual network (3D residual CNN) classifier → DL-ADprob	Non-demented elders; multi-tracer harmonization + prognosis	Harmonized AD amyloid signal improves decline prediction vs raw PET
Sohn et al., 2024	FDG-DL prognosis for pre-dementia AD risk (MCI/CN): incremental value beyond clinical covariates	FDG	FDG-PET_AD pretrained deep CNN→FDG-DL score + saliency maps	ADNI MCI (n = 663), J-ADNI MCI (n = 129), HABS	FDG-DL risk score adds independent prognostic value for AD-related cognitive decline
Ryoo et al., 2024	Subtype discovery / pattern clustering of FDG in the AD spectrum, link subtypes to outcomes	FDG	Conditional variational autoencoder (cVAE) + k-means clustering	Multicenter FDG datasets	Distinct AD-relevant FDG subtypes associate with heterogeneity and clinical outcomes
Park et al., 2023	Tau PET (flortaucipir) classification of AD stages (CN vs MCI vs AD) with multimodal fusion	Tau (flortaucipir)	2D CNN–LSTM and 3D CNN (tau PET), multimodal fusion with clinical/tabular features	ADNI tau PET + clinical/cognitive	Tau-DL improves multi-class AD discrimination, attention maps align with AD topology
Zou et al., 2021	Tau PET DL for cognitive impairment classification; interpretability consistent with Braak-like patterns	Tau (flortaucipir, MK-6240)	InceptionV3-based 2D/3D CNN + saliency	MK-6240 (n = 320) + AV-1451 (n = 446) fivefold CV, cross-tracer evaluation	High performance for impairment classification. Saliency maps highlight Braak-consistent regions and generalize across tau tracers
Kim et al., 2024	Aβ positivity and regional quantification: PET-only vs PET + MRI automated pipelines (Syngo.via vs SCALE PET)	Amyloid (^18^F-flutemetamol)	Logistic regression classifier on regional SUVRs, SUVRs derived via SCALE PET	CABI cohort (n = 1180, 529 Aβ + , 651 Aβ −), no external test	SCALE PET slightly higher overall AUROC for Aβ positivity (0.956 vs 0.947). Strong regional SUVR agreement. PET-only remains competitive
Choo et al., 2025	MRI-free AD amyloid quantification on PET/CT via DL CT parcellation → Centiloid/SUVR	Amyloid (FBB on PET/CT)	FastSurferCNN-inspired multiplanar 2D U-Net ensemble for CT parcellation	Clinical PET/CT, brain pre-contrast CT used to parcellate VOIs	Accurate AD Centiloid/SUVR from PET/CT alone, reduces MRI dependency
Choi et al., 2025	Early-phase amyloid PET + clinical features to predict Aβ positivity and cognitive status for AD screening	Amyloid (early-phase ^18^F-florbetaben)	ML classifiers on early-phase SUVR + clinical data (RF, Grad. Boosting, XGBoost	Clinical dual-phase FBB, model tested on held-out set	Early-phase PET carries AD-relevant predictive signal, supporting faster/leaner screening protocols

**Fig. 3:**
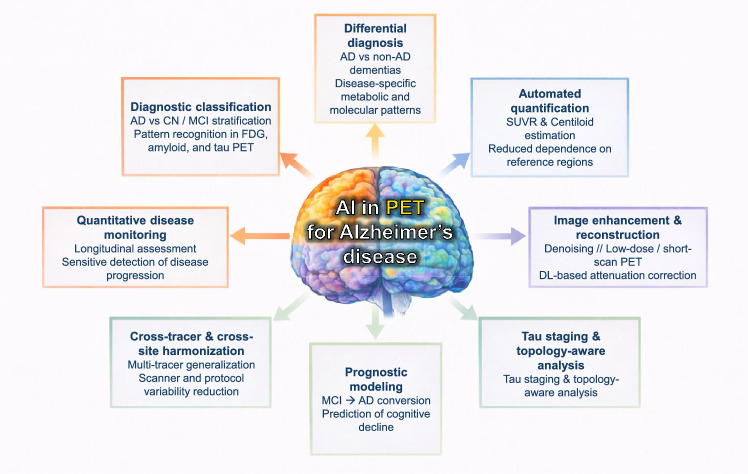
Overview of artificial intelligence applications in PET imaging for Alzheimer’s disease. AI methods are applied across FDG, amyloid, and tau PET to support diagnostic and differential classification, automated quantification (SUVR/Centiloid), image enhancement and reconstruction, cross-tracer and cross-site harmonization, disease staging and longitudinal monitoring, and individualized prognostic modeling

Figure 3 summarizes the main applications of artificial intelligence in PET imaging for Alzheimer’s disease, spanning diagnostic classification and differential diagnosis, automated quantification, image enhancement, cross-tracer and cross-site harmonization, disease staging, longitudinal monitoring, and prognostic modeling across FDG, amyloid, and tau PET.

## Multimodal fusion and cross-modality learning

Fusing PET/SPECT with MRI and clinical/biomarker data generally outperforms single-modality models, provided pipelines are robust to missing data and domain shift. Recent syntheses of PET–MRI DL fusion (early/late/intermediate) show clear gains for AD vs CN/MCI and stress co-registration, resolution mismatch, tracer/site diversity, and external validation [115]. Multimodal systems increasingly favor feature-level (intermediate) fusion with attention/transformers to capture cross-modal dependencies and tolerate absent channels [116, 117].

On the quantitative front, MRI-free amyloid PET quantification is now feasible via DL pre-contrast CT parcellation from PET/CT, enabling Centiloid/SUVR without MRI [118, 119]. In PET/MR, DL attenuation correction has progressed from MRI-based pseudo-CT and direct non-attenuation-corrected (NAC) to AC mapping to clinical evaluation, reducing reconstruction error and simplifying integration when MRI inputs are incomplete [42]. Cross-modality synthesis/supervision (learning one modality from another) further stabilizes multimodal models. Recent surveys and methods describe MRI and PET translation to fill missing channels or regularize representations, with AD-specific PET-from-MRI examples [120, 121]. In the same spirit, DL harmonization across tracers/sites can be folded into fusion pipelines and improves cognitive-decline prediction beyond raw amyloid PET [66].

Beyond imaging-only fusion, hybrid models in Alzheimer’s disease that combine PET (FDG/amyloid/tau) with cognition, demographics, genetics, or plasma biomarkers often enhance prognosis (decline, conversion, time-to-event) and align with calibrated, uncertainty-aware deployment [122].

## Discussion

### Strengths of AI-based SPECT/PET in AD

#### SPECT

In perfusion SPECT (ECD/HMPAO), end-to-end CNN pipelines have shown the ability to classify AD vs cognitively normal and support clinically relevant differential diagnosis (e.g., AD vs VaD/DLB), while providing region-level saliency that can be incorporated into structured reporting [31, 71–73]. Relative to reader-only visual interpretation and template-based z-score heuristics (eZIS/3D-SSP), these models offer data-driven pattern recognition with calibrated probabilities and improved reproducibility, reducing reader dependence and maintaining good performance even in modest SPECT cohorts (e.g., AD vs CN AUC ≈0.90) [71].

Beyond binary decisions, DL feature representations can be mapped to continuous severity surrogates (e.g., MMSE-related indices) to support staging/monitoring and to capture diffuse multiregional topography beyond fixed VOI ratios or single ROI z-scores [71, 73]. When native 3D training volumes are limited, training on template-space z-maps enables learning of disease-specific spatial signatures (e.g., cingulate-island-related patterns in DLB vs AD) while staying close to routine clinical workflows that already generate z-maps and rely on normal databases [74]. In parallel, DL denoising and reconstruction approaches offer a pathway toward low-count [77–82].

#### PET

Deep learning on FDG-PET enables robust discrimination of AD from cognitively normal and supports MCI stratification, with consistently high performance across studies [88]. FDG-based models can also provide prognostic signal for cognitive decline in pre-dementia cohorts, complementing clinical covariates and supporting conversion/decline risk modeling [40, 44, 91]. Established multivariate indices such as the Hypometabolic Convergence Index remain useful quantitative baselines associated with progression in ADNI [92], while network-aware metabolic covariance/graph analyses add systems-level characterization and are increasingly combined with DL for subtyping and representation learning [93, 94].

For amyloid PET, end-to-end native-space models can infer amyloid positivity without MRI and generalize across scanners and tracers at scale, supporting deployable PET-only pipelines [95]. DL also facilitates more automated quantification, including SUVR/Centiloid prediction with reduced preprocessing. For tau PET, DL leverages staged spatial topology for CN/MCI/AD discrimination and yields saliency patterns consistent with Braak-like distributions, supporting biologically plausible interpretation alongside improved multi-class performance [103–105].

### Challenges and methodological considerations of AI in PET/SPECT for AD

Successful translation of AI methods in PET/SPECT to routine AD research and clinical practice remains constrained by reproducibility and generalizability. Domain shift across scanners (and collimators in SPECT), acquisition/reconstruction pipelines, tracers (PET), and site-specific populations can substantially erode performance, while single-center studies may overestimate real-world accuracy. Consequently, external, site-diverse validation with independent test cohorts, confidence intervals, and assessment of clinical utility beyond accuracy is essential [123]. Standardization initiatives partly mitigate variability, yet harmonization must be transparently reported and sensitivity-tested to avoid over-correction where biological differences are treated as batch effects [26, 56, 63–66]. When data sharing is constrained, federated learning can support multi-center development, albeit with added technical and governance requirements [54, 124].

Methodological vulnerabilities also stem from preprocessing assumptions and reference standards. PET quantification choices (e.g., reference region, SUVR/Centiloid implementation) and SPECT reliance on template-space z-maps (eZIS/3D-SSP) can introduce bias. Inaccurate spatial normalization or non-representative normal databases may degrade performance in atypical or mixed-pathology cohorts. Efficiency gains via DL denoising/reconstruction for low-dose/short-scan PET or low-count/short-acquisition SPECT require task-based validation (diagnostic accuracy, SUVR/Centiloid bias, region-wise stability, longitudinal reliability) and safeguards against DL-induced artifacts or systematic bias in quantification [77–82, 98–100]. Finally, clinical trust depends on explainability, calibration/uncertainty estimation, and fairness. Saliency outputs need sanity checks, probabilities should be calibrated, and subgroup performance must be reported to detect hidden stratification, aligned with TRIPOD-AI/CLAIM reporting and emerging regulatory expectations for risk management and lifecycle monitoring [52, 76, 125–129].

### Future perspectives of AI in SPECT/PET for AD

Future AI development in PET/SPECT is likely to shift from single-center diagnostic demonstrations toward standardized, generalizable, and longitudinally reliable decision support across tracers and sites. For PET, this includes scalable native-space, MRI-free classification/quantification, stronger cross-tracer transfer anchored to Centiloid/EARL-aligned harmonization, and broader use of prognostic and time-to-event modeling that integrates imaging with clinical, cognitive, and blood biomarkers. For SPECT, emphasis will be on consolidating perfusion SPECT as a widely accessible quantitative biomarker through multi-center validation, harmonization across cameras/ collimators/reconstruction, and careful task-based qualification of DL-enabled low-count/short-scan protocols for safer follow-up. Across both modalities, self- or weakly-supervised pretraining, federated multi-center learning, and explicit calibration and uncertainty reporting are poised to improve robustness in heterogeneous populations, while adherence to reporting standards and regulatory-aligned lifecycle monitoring will be key for clinical adoption and sustained performance over time [61, 88, 124, 127, 129].

## Conclusions

AI methods are increasingly used to support quantitative analysis in PET and SPECT for Alzheimer’s disease, with demonstrated utility in classification, differential diagnosis, and tracer-specific quantification. The field’s impact will be determined less by incremental gains in headline accuracy and more by whether models can deliver reliable, comparable outputs across sites, scanners, and tracers, and whether those outputs remain stable in longitudinal use and heterogeneous real-world cohorts.

Accordingly, the central requirement for translation is methodological: standardization and harmonization, site-diverse external validation with calibration/uncertainty, and transparent reporting that enables reproducibility and subgroup accountability. If these criteria are met, AI-enabled PET/SPECT can pragmatically support earlier triage, more consistent differential diagnosis, and scalable quantitative monitoring, especially when integrated with clinical/cognitive and emerging blood biomarkers, while maintaining human oversight and lifecycle monitoring consistent with evolving regulatory expectations.

## Data Availability

This article is a review and does not report original data. No new data were generated or analyzed, therefore, data sharing is not applicable.
